# Social vulnerability amplifies the disparate impact of mobility on COVID-19 transmissibility across the United States

**DOI:** 10.1057/s41599-022-01437-5

**Published:** 2022-11-24

**Authors:** Bo Huang, Zhihui Huang, Chen Chen, Jian Lin, Tony Tam, Yingyi Hong, Sen Pei

**Affiliations:** 1 Department of Geography and Resource Management, The Chinese University of Hong Kong, Hong Kong, Hong Kong SAR; 2 Department of Sociology, The Chinese University of Hong Kong, Hong Kong, Hong Kong SAR; 3 Faculty of Geosciences and Environmental Engineering, Southwest Jiao Tong University, Chengdu, China; 4 Sierra Nevada Research Institute, University of California Merced, Merced, USA; 5 Department of Management, The Chinese University of Hong Kong, Hong Kong, Hong Kong SAR; 6 Department of Environmental Health Sciences, Mailman School of Public Health, Columbia University, New York, NY 10032 USA

**Keywords:** Geography, Social policy

## Abstract

Although human mobility is considered critical for the spread of the new coronavirus disease (COVID-19) both locally and globally, the extent to which such an association is impacted by social vulnerability remains unclear. Here, using multisource epidemiological and socioeconomic data of US counties, we develop a COVID-19 pandemic vulnerability index (CPVI) to quantify their levels of social vulnerability and examine how social vulnerability moderated the influence of mobility on disease transmissibility (represented by the effective reproduction number, *R*_t_) during the US summer epidemic wave of 2020. We find that counties in the top CPVI quintile suffered almost double in regard to COVID-19 transmission (45.02% days with an *R*_t_ higher than 1) from mobility, particularly intracounty mobility, compared to counties in the lowest quintile (21.90%). In contrast, counties in the bottom CPVI quintile were only slightly affected by the level of mobility. As such, a 25% intracounty mobility change was associated with a 15.28% *R*_t_ change for counties in the top CPVI quintile, which is eight times the 1.81% *R*_t_ change for those in the lowest quintile. These findings suggest the need to account for the vulnerability of communities when making social distancing measures against mobility in the future.

## Introduction

The new coronavirus disease (COVID-19) pandemic has caused profound disruptions to the lives of people worldwide (Bonaccorsi et al., 2020; Buckee et al., 2021) and disproportionately affected disadvantaged and underprivileged subpopulations (UN, 2020; Buckee et al., 2021). The devastating economic and social effects caused by the pandemic necessitate an investigation into the drivers of disease transmission in the past to formulate appropriate and effective preventive strategies in the future.

As the major routes of transmission for severe acute respiratory syndrome coronavirus 2 (SARS-CoV-2) are via direct physical contact, droplets, or aerosols, the human movement has been considered critical for the spatial and temporal spread of the disease (Kraemer et al., 2020; Huang et al., 2021). That is, mobility directly contributes to the dispersal of infections through social contact. However, different social groups in terms of income, employment status, and/or age may be vulnerable to the disease to varying degrees due to their mobility abilities and patterns, behaviours and lifestyles, and socioeconomic resources. Thus, the interplay between social vulnerability, mobility, and transmission is complex, and there is an urgent need to understand their interrelationships to make more pertinent public health and social measures against future waves of COVID-19 and other public health crises.

Since the initial outbreak of COVID-19 in Wuhan, China, a plethora of research has assessed the influence of mobility on COVID-19 transmission using anonymized aggregate mobile phone data (Buckee et al., 2020; Kraemer et al., 2020; Tian et al., 2020). The impact of mobility on COVID-19 transmission rates in 25 counties in the US has also been evaluated (Badr et al., 2020), and a strong correlation between mobility patterns and COVID-19 growth rates in the most affected United States (US) counties was found. Similarly, a strong correlation was found at the state level in the US (Gao et al., 2020). These results explain why mobility reduction, such as stay-at-home orders, the closure of schools and workplaces, and restrictions on long-distance travel, was adopted as a primary nonpharmaceutical intervention (NPI) to contain the transmission of the disease. As of July 2021, 186 countries had announced at least one domestic mobility control policy, while 186 countries had also employed an international policy (Hale et al., 2021). These mobility control measures achieved considerable effects, as transmission decreased in 73% of the countries following their first mobility interventions (Nouvellet et al., 2021). Based on cell phone location data from Shenzhen, China, changes in COVID-19 transmission during the course of reopening were simulated by varying the type of mobility restrictions under different transmission scenarios; it was found that a 20–60% reduction in mobility within the city had a significant effect on controlling the spread of COVID-19 (Zhou et al., 2020).

Although an extensive body of literature has investigated the impact of mobility on transmission dynamics, the extent to which such an association is moderated by social vulnerability and varies across geographical areas and different social groups remain largely unexplored. The majority of studies focus on the relationships between vulnerability and mobility and between vulnerability and disparate pandemic outcomes, e.g., mobility reduction as related to income during the lockdown period (Bennett, 2021; Hou et al., 2021), infection risk as related to income (Rufat et al., 2015; Eligon et al., 2020; Snyder and Parks, 2020; Cahill et al., 2021), and the age structure as related to infection or mortality rates (Gu et al., 2020; Snyder and Parks, 2020). However, these studies do not address the varying impacts of mobility on transmission dynamics due to social vulnerability, i.e., how social vulnerability disparately affects the mobility–transmission association. It has been found to be inappropriate and insufficient to apply a uniform relationship between mobility and transmission across counties and social groups with disparate socioeconomic statuses when formulating preventive measures (Gozzi et al., 2021). Understanding such heterogeneous effects can help policymakers target certain social groups and thus make more effective interventions to mitigate COVID-19 transmission and ameliorate social inequity. Moreover, social vulnerability is a multiple-dimensional concept that is rooted in the interactions among social, natural, and engineered systems (Cutter, 2003). With the notion of this complexity, social vulnerability is typically conceptualized as consisting of different dimensions; in practice, these dimensions are collapsed into composite indicators such as the Social Vulnerability Index (SoVI) (Spielman et al., 2020). However, most of the existing studies select a single or a small subset of sociodemographic variables to explore COVID-19 transmission among different vulnerable communities, thereby neglecting the combined effect of multiple variables on COVID-19 transmissibility (Fauver et al., 2020; Tian et al., 2020; Xu and Li, 2020; Cahill et al., 2021). Thus, a comprehensive measure of COVID-19 vulnerability is needed to better understand the socially heterogeneous mobility–transmission relationship.

Efforts have been made to construct a multidimensional COVID-19 vulnerability index (see a more detailed review in the Supplementary Materials). For example, one group of studies follows the traditional framework of social vulnerability to select relevant variables (Acharya and Porwal, 2020; Kim and Bostwick, 2020; Macharia et al., 2020; Snyder and Parks, 2020; Daras et al., 2021; Sarkar and Chouhan, 2021; Qiao and Huang, 2022; Welsh et al., 2022), with the aim of examining the explanatory power of the framework or certain variables. In contrast, another group of studies attempts machine learning methods to assess the vulnerability of administrative units. For instance, Tiwari and colleagues (2021) created the COVID-19 pandemic vulnerability index using random forests and then classified counties in the United States into varying levels of vulnerability. Compared to the traditional methods, the indices constructed via machine learning are less restricted by the conventional theories present in statistics and may more accurately predict the dynamics of the COVID-19 pandemic in communities. However, the advantages of such data-driven methods could become constraints when testing theoretical hypotheses and the internal consistency of the index.

This study investigates COVID-19 transmissibility across counties in the US affected by mobility changes following successive reopening policies implemented by state and local governments. We first develop a COVID-19 pandemic vulnerability index (CPVI) for US counties using their census data via traditional principal component analysis (PCA). Our CPVI is built on the well-established framework of the SoVI from the Centers for Disease Control and Prevention (CDC) (CDC, 2020). In addition to the four dimensions of the SoVI, other factors relevant to the pandemic, i.e., health and the environment, are also included because several studies have found various influences of these factors on COVID-19 spread (Chin et al., 2020; Klompmaker et al., 2021; Marvel et al., 2021). Based on the resulting CPVI, the counties are then divided into five quintiles, with the top 20% representing the most vulnerable group of counties. By including the interaction of mobility and vulnerability in a fixed effect model, the heterogeneous effect of mobility on COVID-19 transmission under varying vulnerability levels (corresponding to the five quintiles) is then observed. In addition, the differences in the effect of vulnerability levels on transmissibility, as represented by the effective reproduction number (*R*_t_), are estimated and tested. The empirical results show that mobility reduction measures can be implemented in a manner that is more pertinent with respect to a county’s vulnerability level, thereby facilitating a more effective containment of COVID-19 infections.

## Methods

### COVID-19 cases

Statistical data on COVID-19 and the population were collected from USAFacts, which is used by the US CDC. The dataset contains the cumulative number of daily confirmed cases and the cumulative number of COVID-19 deaths in every county in the US. We downloaded the case data from January 22, 2020, to September 1, 2020, and then we derived the number of new cases each day within the period.

### County attribute data

The county attribute data were obtained from multiple publicly available data sources. First, data on demographics (e.g., the percentages of the elderly population and ethnic minority population), socioeconomics (e.g., income and poverty), diseases (e.g., diabetes and hypertension data), and health insurance were retrieved from the CDC’s 2015–2018 statistical dataset. Second, the air pollution data were collected from the County Health Ranking & Roadmaps, which count the yearly average PM_2.5_ concentration for each county. Third, we collected 2017 data on airborne isolation rooms and hospital bed capacity from the Area Health Resources Files. Finally, the normalized difference vegetation index (NDVI) was retrieved from the Moderate Resolution Imaging Spectroradiometer (MODIS) global vegetation index data product (MOD13A2), which provides global gridded NDVI products. The average NDVI values of each county in 2019 were later calculated using the Google Earth Engine (GEE). These metrics were selected because extensive studies have found that they have differential effects on the spread of epidemics (Baron, 2020; Onder et al., 2020, Tahmasebi et al., 2020; Yan et al., 2020; Zoran et al., 2020; Klompmaker et al., 2021). Table S1 lists all the indicators and relevant information used.

### Population mobility data

Data on population mobility were originally collected by SafeGraph, which tracks the trajectory of millions of anonymous mobile phone users and generates a daily human movement origin-to-destination (O–D) flow matrix at the county scale in the US. We obtained the O–D data for the period of January 1, 2020–September 1, 2020, and these data were further processed and made available by Kang et al. (2020).

With the O–D dataset, we obtained the inflow population (Inflow^*t*^), outflow population (Outflow^*t*^), and internal mobile population (Intraflow^*t*^) for each county on day *t*. We took the average population mobility from January 1, 2020, to January 21, 2020, prior to the outbreak in the US as the baseline. Thus, the changes in IntraM for each county during the outbreak can be expressed as follows:

1 $${\rm {IntraM}}_i^t = \frac{{{\text {Intraflow}}_i^t}}{{{\text {Intraflow}}_i^{t_0}}}$$

where ${\text {IntraM}}_i^t$ denotes the daily change rate of IntraM on day *t*, ${\text {Intraflow}}_i^t$ denotes the IntraM on day *t*, and ${\text {Intraflow}}_i^{t_0}$ denotes the daily average of IntraM during the baseline period. All for county *i*. IntraM^*t*^ > 1 indicates an increase of IntraM relative to the baseline period, whereas IntraM^*t*^ < 1 indicates a decrease.

Similarly, we also calculated the daily change rate of InterM for each county, which can be expressed as follows:

2 $${\text {InterM}}_i^t = \frac{{{\text {Inflow}}_i^t + {\text {Outflow}}_i^t}}{{{\text {Inflow}}_i^{t_0} + {\text {Outflow}}_i^{t_0}}}$$

where ${\text {InterM}}_i^t$ denotes the change rate of InterM on day *t* for county *i*. The numerator denotes the sum of the inflow and outflow of county *i* on day *t*, and the denominator denotes the daily average of the sum of the inflow and outflow of county *i* during the baseline period.

### Setting and selection of study participants

The US directives to shelter in place and temporarily close nonessential businesses and schools were made at the state and local levels throughout March and April 2020 (Badr et al., 2020). The national average mobility then decreased rapidly, followed by a slow decrease in daily new cases. However, in late April and May, the states enacted additional reopening policies, leading to the gradual recovery of mobility (Smith et al., 2020). At the beginning of the outbreak, many studies demonstrated that there was a strong correlation between the spread of COVID-19 and population movement (Badr et al., 2020; Coelho et al., 2020, Rubin et al., 2020; Chang et al., 2021; Nouvellet et al., 2021). However, with the reopening of the states, mobility started to recover, and the number of infections also rebounded rapidly, ushering in a second wave of the pandemic (Fig. S1). While many studies have examined mobility in relation to COVID-19 transmission during the prelockdown and lockdown periods, few studies have explored this relationship postlockdown without vaccination. Therefore, we chose the period of June 1, 2020–August 31, 2020, for our analysis, centring on the mobility–transmissibility relationship.

We set the following county-filtering criteria: counties in the contiguous US, excluding Alaska and Hawaii; counties that had at least one case of COVID-19 as of June 1, 2020; and counties that did not have an average of fewer than three cases per day ranging from June 1, 2020, to August 31, 2020. Counties with a 3-day average of less than one case were excluded. Ultimately, 1118 (out of 3143) counties were selected, covering 257,867,883 people or 78.56% of the total US population, and the cumulative number of confirmed cases in these counties as of September 1, 2020, was 4,980,400 or 82.75% of the total. These counties are distributed in 46 US states and Washington, DC.

### Construction of the CPVI

There have been several attempts to construct a COVID-19 vulnerability index (Acharya and Porwal, 2020; Marvel et al., 2021; Tiwari et al., 2021). The basic strategy is a combination of the SoVI used by the US CDC and other factors closely related to the COVID-19 pandemic, such as epidemiological factors and healthcare system factors.

Our CPVI is built on previous social vulnerability indices by selecting the four dimensions used by the SoVI (Spielman et al., 2020) and incorporating epidemiological and healthcare system factors. Eight out of 15 SoVI variables (being an elderly individual, ethnicity, groups, language, lower education attainment, income, poverty, and unemployment) and 9 epidemiological and healthcare system variables (smoking, diabetes, coronary heart diseases, hypertension, air pollution, green exposure, isolation rooms, hospital beds, and insurance) were included in the calculation after selection based on the previous literature (Supplemental Materials). This process allows us to estimate the contribution of each indicator and component and thus test the internal consistency and reliability of the CPVI (Spielman et al., 2020).

The widely used PCA with a varimax rotation matrix is adopted to eliminate redundant information and construct a set of PCs (Halko et al., 2011). The PCs with eigenvalues greater than one should be retained, as each of them, explains more variance than a single variable in the original data (Bro and Smilde, 2014). The loadings of the rotation factors are used to calculate the scores of each PC on the samples. The score vector of the *k*th PC on the sample is denoted as *S*_*i*_; then, it can be expressed as follows:

4 $$S_j = Xa_j^{\text {T}}$$

where *X* denotes the matrix formed by the values of each indicator in each sample and *a*_*j*_ is the factor loading vector of the *j*th principal component.

The contribution weights of each subcomponent are calculated by their contribution proportion as follows:

5 $$W_j = \frac{{p_j}}{{\mathop {\sum}\nolimits_{j = 1}^m {p_j} }}$$

where *W*_*j*_ denotes the weight of the *j*th subcomponent, *m* denotes the number of subcomponents, and *p*_*j*_ denotes the proportion of the contribution of the *j*th subcomponent.

Finally, the CPVI is calculated as follows:

6 $${\text {CPVI}} = \mathop {\sum}\limits_{j = 1}^m {S_jW_j}$$

The CPVI is divided into five quintiles, with the first quintile (level 1) indicating the lowest vulnerability level and the last quintile (level 5) indicating the highest vulnerability level. Each county is assigned a specific vulnerability level based on its CPVI value.

### Calculation of *R*_*t*_

Similar to other infectious diseases (e.g., severe acute respiratory syndrome (SARS) and influenza), the real-time transmissibility of COVID-19 can be estimated using the effective reproduction number (*R*_*t*_) (Cauchemez et al., 2006; Cowling et al., 2020). *R*_*t*_ is the average number of secondary cases per case at any given time, and it provides a useful measure of how quickly the virus is spreading. As the pandemic progresses, implementing various restrictions and interventions will change the *R*_*t*_ value. If *R*_*t*_ is >1, each infected case will go on to infect an average of more than one person, and the number of infected cases may increase exponentially. If *R*_*t*_ is <1, each infected case will go on to infect an average of less than one person, possibly suggesting a slowdown of the outbreak over time.

We use the approach developed by Bettencourt and Ribeiro (2008) to calculate the *R*_*t*_ of US counties (Bettencourt and Ribeiro, 2008). The daily additions of confirmed cases provide information on the current value of *R*_*t*_; hence, *R*_*t*_ can be estimated using such numbers. In addition, the value of *R*_*t*_ for the current day is related to the values of *R*_*t*_ one day before and every previous day since the outbreak. The approach is based on the Bayesian framework to estimate the value of the daily *R*_*t*_ using the new cases reported daily:

7 $$P\left( {R_t\left| k \right.} \right) = \frac{{P\left( {k\left| {R_t} \right.} \right)P\left( {R_t} \right)}}{{P\left( k \right)}}$$

where *P*(*k*|*R*_*t*_) is the likelihood of observing *k* new cases given *R*_*t*_ on day *t*, *P*(*R*_*t*_) is the prior beliefs of the value of *P*(*R*_*t*_) at the beginning of the study period, and *P*(*k*) is the probability of observing *k* new cases for the given day *t*.

Given an average arrival rate of *λ* new cases per day, the probability of observing *k* new cases follows the Poisson distribution:

8 $$P\left( {k{{{\mathrm{|}}}}\lambda } \right) = \frac{{\lambda ^ke^{ - \lambda }}}{{k!}}$$

Then, the relationship between *R*_*t*_ and *λ* exists as follows:

9 $$\lambda = k_{t - 1}{\text {e}}^{L\left( {R_t - 1} \right)}$$

where *L* is the reciprocal of the serial interval, and the value of the serial interval is assigned with the mean (standard deviation) (i.e., 7.5 (3.4) days) according to a previous epidemiological survey (Li et al., 2020; Rubin et al., 2020). Furthermore, since the number of daily new cases is known, the Poisson parameterized likelihood function can be re-expressed by fixed *k* and varying *R*_*t*_ (48):

10 $$P\left( {k{{{\mathrm{|}}}}R_t} \right) = \frac{{\lambda ^ke^{ - \lambda }}}{{k!}}$$

### Fixed effect model

We formulate a fixed effect model to estimate the heterogeneous association between mobility and transmission based on the county-level CPVI value divided into five vulnerability levels.

The regression of mobility and transmission at time *t* is written as follows:

11 $$\begin{array}{l}y_{it} = \beta _0 + \beta _1{\rm {IntraM}}_{it} + \beta _2{\rm {InterM}}_{it} + \beta _3{\rm {CPVI}}_i + \beta _4{\rm {IntraM}}_{it} \\ \qquad*\, {\rm {CPVI}}_i + \beta _5{\rm {InterM}}_{it} \,*\, {\rm {CPVI}}_i + \beta _6Z_i + \mu _{it}\end{array}$$

where *y*_*it*_ is the dependent variable denoting COVID-19 transmissibility (*R*_*t*_). IntraM_*it*_ and InterM_*it*_ denote the two mobility measurements, namely, IntraM and InterM, respectively; CPVI_*i*_ denotes the COVID-19 pandemic vulnerability index value; the interactions between the mobility measurements and vulnerability (IntraM_it_*CPVI_*i*_ *and* InterM_it_*CPVI_*i*_) denote the moderating effects of vulnerability; *Z*_*i*_ denotes other observable and unobservable time-invariant factors (except vulnerability) affecting COVID-19 transmissibility; and *μ*_*it*_ is the time-specific and individual-specific residual. *βs* are the regression coefficients, *β*_1_ and *β*_2_ are the coefficients of the main effect of mobility on transmission, and *β*_4_ and *β*_5_ denote the magnitude of the moderating effect of vulnerability.

Take the average of both sides as follows:

12 $$\begin{array}{l}\bar y_i = \beta _0 + \beta _1\overline {\rm {{IntraM}}} _i + \beta _2\overline {\rm {{InterM}}}_i + \beta _3{\rm {CPVI}}_i + \beta _4\overline {{\rm {IntraM}}} _i\\ \qquad*\, {\rm {CPVI}}_i + \beta _5\overline {{\rm {InterM}}} _i \,*\, {\rm {CPVI}}_i + \beta _6Z_i + \overline \mu _i\end{array}$$

The difference between Eqs. (11) and (12) are as follows:

13 $$\begin{array}{l}y_{it} - \overline y _i = + \beta _1\left( {{\rm {IntraM}}_{it} - \overline {{\rm {IntraM}}} _i} \right) + \beta _2\left( {{\rm {InterM}}_{it} - \overline {{\rm {InterM}}} _i} \right)\\ \qquad+\, \,\beta\, _4{\rm {CPVI}}_i * \left( {{\rm {IntraM}}_{it} - \overline {{\rm {IntraM}}} _i} \right) + \beta _5{\rm {CPVI}}_i \\\qquad*\, \left( {{\rm {InterM}}_{it} - \overline {{\rm {InterM}}} _i} \right) + \left( {\mu _{it} - \overline \mu _i} \right)\end{array}$$

Alternatively:

14 $$\begin{array}{l}\widetilde y_i = \beta _1\widetilde {\rm {{IntraM}}}_{it} + \beta _2\widetilde {{\rm {InterM}}}_{it} + \beta _4{\rm {CPVI}}_i \,*\, \widetilde {{\rm {IntraM}}}_{it}\\ \qquad+\, \beta _5{\rm {CPVI}}_i \,*\, \widetilde {{\rm {InterM}}}_{it} + \widetilde \mu _{it}\end{array}$$

Therefore, all the time-invariant factors, including the COVID-19 vulnerability index CPVI_*i*_ and other observed and unobserved factors *Z*_*i*_, are differenced out. Only the difference in transmission, the difference in mobility measurements, and the interactions are left.

## Results

### COVID-19 pandemic vulnerability index

Tables 1 and S1 list the data collected to build the CPVI. The data cover the demographics, social economy, disease, natural environment, and medical security of the counties in the US. After conducting PCA (see the details in the “Methods” section), four meaningful components explaining over 70% of the total variance in the dataset were derived (Table 1). Given the large deviations in the vulnerability variables of some counties, logarithmic operations were performed and then standardized using a mean-standard deviation function. Positive directionalities were assigned to the loadings of principal components (PCs) that were observed to increase the COVID-19 pandemic, whereas negative directionalities were assigned to the loadings of PCs that were observed to decrease the COVID-19 pandemic.

**Table 1:** Principal component analysis results.

PCs	% of variance	Variables	Component loading
PC1	38.58	Unemployment	0.976
		Poverty	0.970
		Ethnicity	0.974
		Elderly individual	0.935
		Lower education attainment	0.970
		Language	0.897
		Groups	0.822
PC2	16.63	Air pollution	0.654
		Green exposure	−0.642
		Hypertension	0.563
		CHD	0.565
		Diabetes	0.500
		Smoking	0.654
PC3	11.79	Income	−0.734
		Insurance	0.546
PC4	8.30	Isolation rooms	−0.694
		Hospital beds	−0.703

The PCA uncovered four components with eigenvalues >1.0. PC1 explains 38.58% of the total variance and consists of unemployment, poverty, ethnicity, being an elderly individual, lower education attainment, language, and groups. As a result, PC1 is positively correlated with all seven variables. PC2, which explains 16.63% of the total variance in the dataset, is a component that is positively correlated with air pollution, hypertension, coronary heart disease (CHD), diabetes, and smoking but negatively correlated with green exposure. Accounting for 11.79% of the total variance in the dataset, PC3 is negatively correlated with income and positively correlated with insurance. Finally, PC4, which explains 8.30% of the total variance in the dataset, is negatively correlated with isolation rooms and hospital beds.

**Fig. 1:**
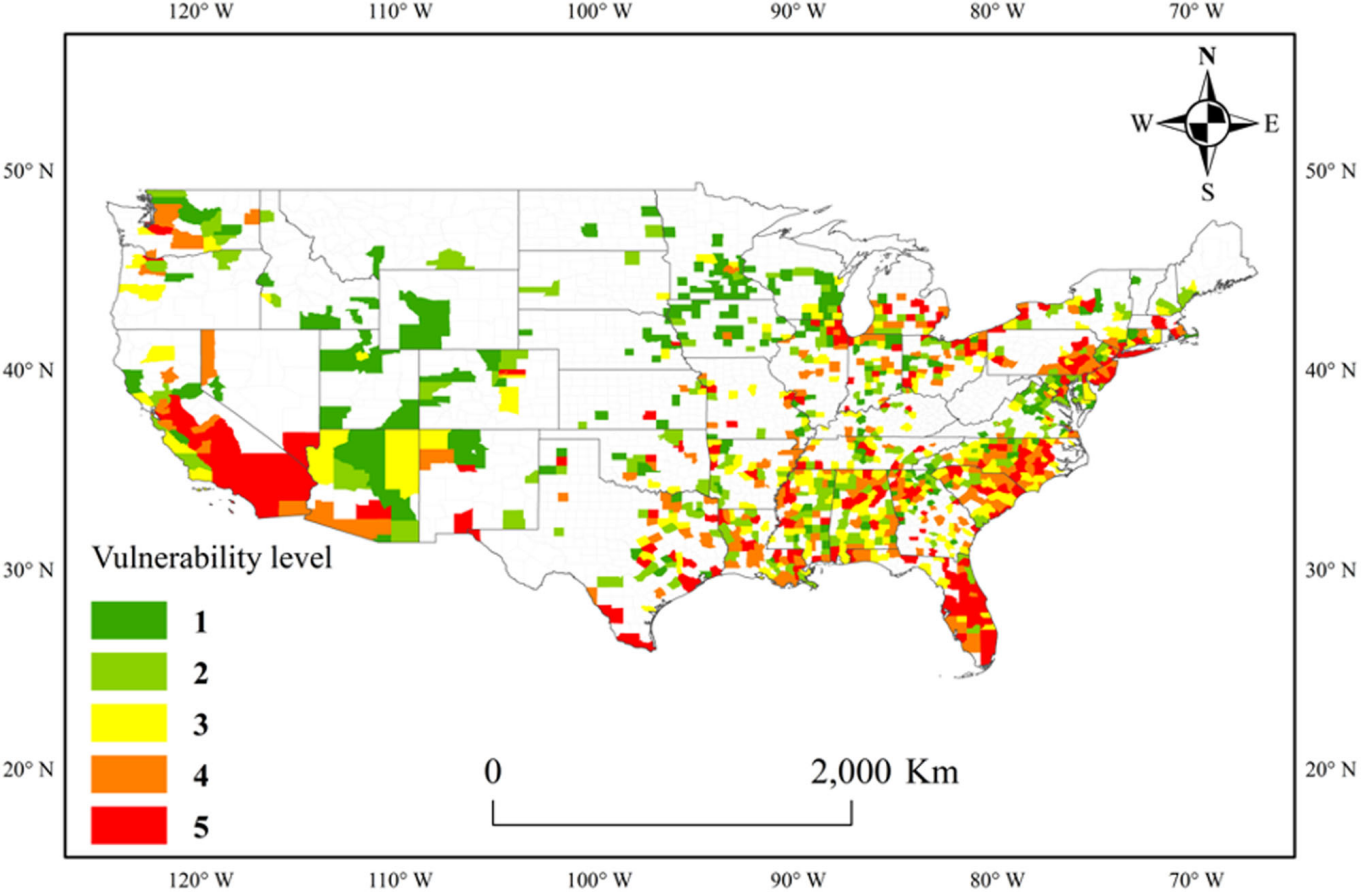
Spatial distribution of counties with different vulnerability levels, as measured by the CPVI. The CPVI values are divided into five levels based on quintiles, where 5 indicates the most vulnerable communities and 1 indicates the least vulnerable communities. White areas indicate counties where no data are available.

The CPVI of each county was calculated by weighting based on the variance ratio of each PC. Then, the CPVI values were divided into five vulnerability levels by quintile. The selected set of counties and their corresponding vulnerability levels are displayed in Fig. 1. This map captures the counties with the highest vulnerability levels for a variety of reasons: high unemployment rates and poverty rates (e.g., in Mississippi and South Carolina), the counties with the most minorities and the worst air quality (e.g., in California), the counties with more elderly people (e.g., in Florida, North Carolina and Alabama), the counties with more chronic diseases (e.g., in New York), and the counties with bad air quality and medical resource shortages (e.g., in the Great Lakes coastal region). In contrast, in the counties with lower air pollution and a younger age structure, such as those in the Central Plain, the CPVI is lower. This pattern is consistent with the COVID-19 Pandemic Vulnerability Index Dashboard Map (date: 01/07/2020) released by the National Institute of Environmental Health Science.

The distribution of the CPVI is not identical to any of the characteristics included. For example, counties in California have a higher personal income and level of education than the US average; thus, they should be considered to have the lowest vulnerability if sorted solely by socioeconomic characteristics. However, they are identified as having a vulnerability level of 5, which is the highest vulnerability level. Similarly, Florida is equipped with good medical resources but still has many counties that are identified as having a vulnerability level of 4 or 5. This finding shows that the CPVI does not sort counties based solely on one dimension, such as socioeconomic or pandemic factors, and is thus able to capture the combination of dimensions.

### Variation in COVID-19 transmissibility across different vulnerability levels

**Fig. 2:**
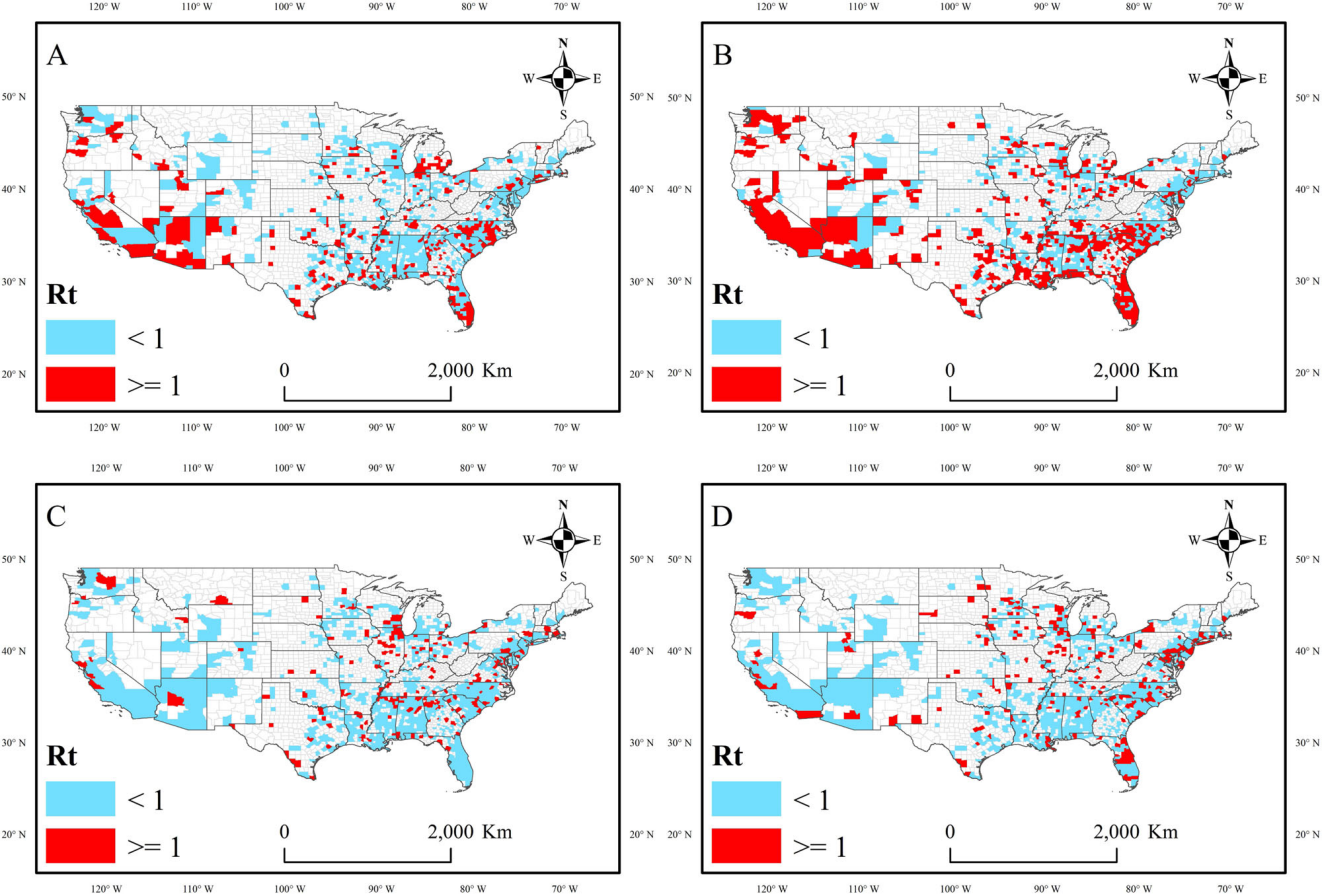
Spatial distribution of Rt values by county at four time points. We take 1 as the standard value and divide those with an *R*_*t*_ ≥ 1 into one category (marked in red) and those with an *R*_*t*_ < 1 into another category (marked in blue). **A** June 1, 2020, **B** July 1, 2020, **C** August 1, 2020, and **D** August 31, 2020. White areas indicate counties where no data are available.

In this study, the effective reproduction number (*R*_*t*_) represents the daily transmissibility of COVID-19 (see more details about how to derive it in the “Methods” section). Figure 2 shows the change in *R*_*t*_ for each county on the 4 selected days (June 1, July 1, August 1, and August 31) from June 1 to August 31, 2020. On June 1, 2020, 310 counties had an *R*_*t*_ ≥ 1 (highly transmissible), representing 36.85% of the counties under study (i.e., those for which the *R*_*t*_ can be derived). Of these 4 days, there was the largest number of counties with an *R*_*t*_ ≥ 1 (i.e., 559 counties) on July 1, 2020; this total is more than 50% of the counties under study. The distribution of the hardest-hit counties on June 1 and July 1, 2020, coincided with that of the counties with high CPVI values (Fig. 2A and B) and was concentrated in the coastal areas of California, Florida, and some east-central states. The numbers and distributions of counties with high transmissibility on August 1 and August 31 were similar, i.e., 227 and 271, respectively. They were mainly located in the central-east states, with fewer in the west (Fig. 2C and D).

We calculated the *R*_*t*_ for 102,856 county days (1118 counties * 92 days) from June 1 to August 31, 2020. A county day is defined as a highly transmissible day if its *R*_*t*_ is ≥1 on that day. To explore transmissibility in relation to vulnerability during the study period, we counted the total number of highly transmissible days at different vulnerability levels. Table 2 shows the percentage of highly transmissible days of all county days in the counties under study. When aggregated at the monthly level, the percentage of highly transmissible days increased from June to July and then decreased from July to August. Such a sequence of change is relatively close to the overall US pandemic trend (Fig. S1). In terms of the vulnerability level in each month and overall, the percentage of highly transmissible days increased as the vulnerability level rose. In particular, the number of level 5 locations remained above 50% in both June and July (53.66% in June and 58.72% in July).

**Table 2:** Percentage of days (%) with an *R*_*t*_ ≥ 1 at different vulnerability levels.

Month	Vul. level				
	1	2	3	4	5
June	21.73	29.10	39.37	43.38	53.66
July	30.74	39.90	49.15	54.41	58.72
August	13.22	15.62	16.69	22.11	22.96
Total	21.90	28.19	36.30	39.93	45.02

Vul. level stands for vulnerability level.

**Fig. 3:**
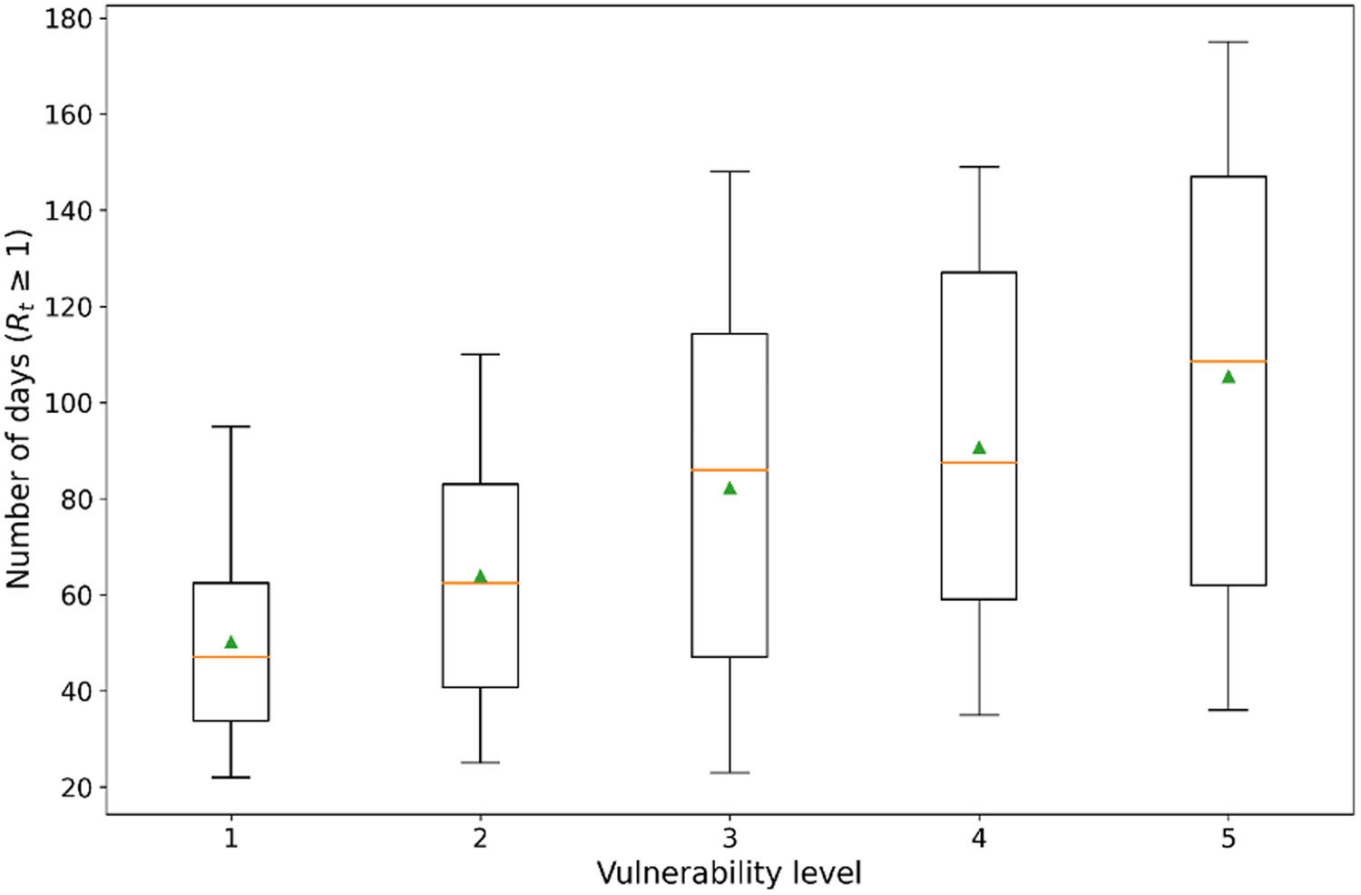
Changes in the number of highly transmissible days with varying vulnerability levels. The solid brown line indicates the median value, and the green triangle indicates the mean value.

Figure 3 shows a comparison of the number of highly transmissible days at each vulnerability level from June 1 to August 31. There was a positive correlation found between COVID-19 transmissibility and vulnerability level. In addition, the fluctuation in the number of county days with high transmissibility per day increased with the increase in the vulnerability level.

### Variations in mobility recovery across different vulnerability levels

**Fig. 4:**
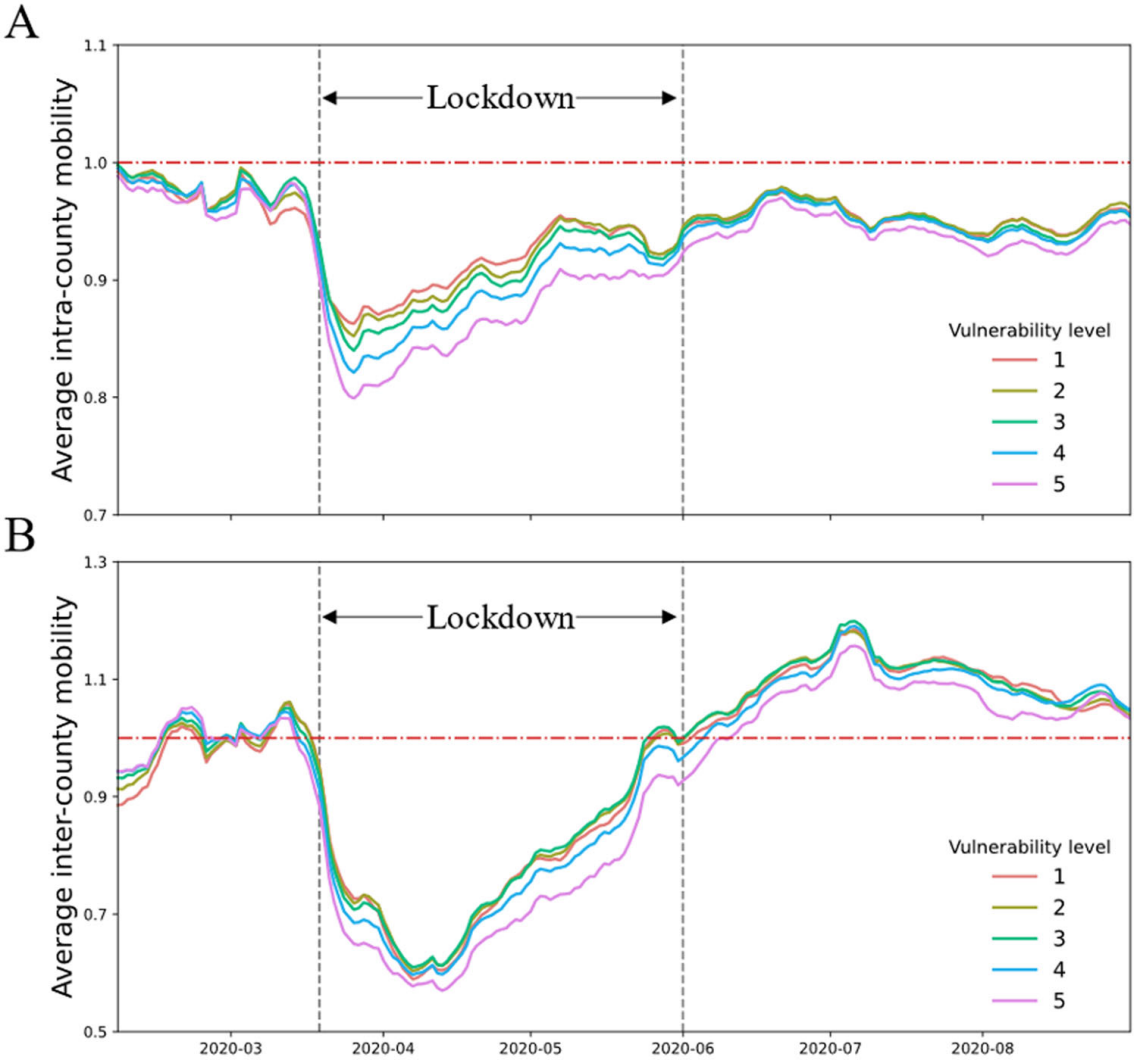
Temporal variation in mobility across different vulnerability levels. Baseline value 1 is marked in red in the figure, and all mobility values are smoothed using a 7-day moving average. **A** The average changes in IntraM. **B** The average changes in InterM.

We differentiated intracounty mobility (IntraM) from intercounty mobility (InterM) and plotted the average values of these two indices for each vulnerability level (Fig. 4). The indices displayed a very consistent pattern in which the values decreased and recovered. With the IntraM from the prelockdown period falling to its lowest level on March 21 during the lockdown period and after reopening, there was a consistent inverse relationship between IntraM and the vulnerability level; i.e., the higher the vulnerability level is, the lower the IntraM is (Fig. 4A). For varying vulnerability levels, the drops in IntraM to the bottom were different. The group of counties with the lowest level of vulnerability showed a decrease of 24.94% relative to their baselines, whereas the decrease was 32.98% for the group with the highest level of vulnerability; thus, the latter decreased by 8.04% more than the former.

On the other hand, the InterM from the prelockdown period dropped to its lowest level on April 12. The group of counties with the lowest vulnerability showed a decrease of 62.72%, whereas the group with the highest vulnerability showed a decrease of 66.36%. The latter decreased by 5.50% more than the former. Another interesting aspect is that before the lockdown period, InterM was higher in more vulnerable groups of counties than in less vulnerable groups; however, during and after the lockdown period, the opposite trend was observed (Fig. 4B).

In this paper, mobility (including both IntraM and InterM) is defined as a ratio relative to the average mobility during the normal period in the US, January 1 to 21, 2020 (see more details in the “Methods” section). A higher (lower) mobility during and after the lockdown period indicates a higher (lower) recovery of mobility. With this notion in mind, the empirical interpretation of the relationship between vulnerability and mobility is as follows: even though the average IntraM (and InterM) of all 5 vulnerability levels fluctuate in a similar pattern, the higher the vulnerability level is, the lower the recovery level of mobility is.

### The effect of mobility on *R*_*t*_ under varying vulnerability levels

**Fig. 5:**
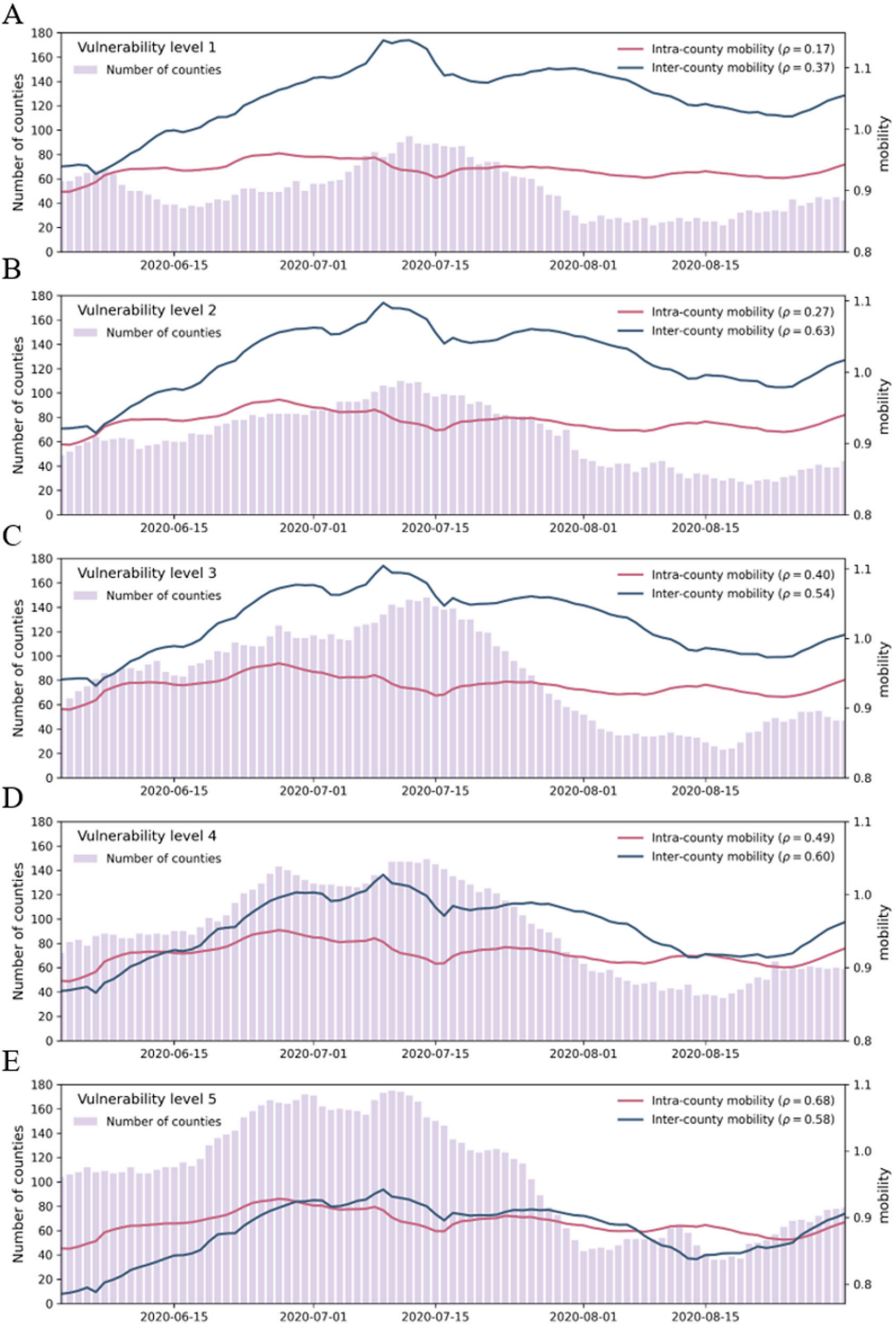
The average mobility and number of counties per day with a daily *R*_*t*_ ≥ 1 (i.e., counties with high transmissibility) across varying vulnerability levels from June 1 to August 31. **A**–**E** vulnerability levels 1–5. Mobility includes both IntraM and InterM. All mobility values are the average of each level and smoothed using a 7-day moving average. All Pearson correlation coefficients between mobility and the number of counties with high transmissibility are statistically significant (*p* < 0.05).

To examine the relationship between mobility and COVID-19 transmissibility in counties with different levels of vulnerability, we calculated the correlations of IntraM and InterM with high transmissibility days for all counties together and for counties in separate vulnerability groups. For all counties, the correlations of IntraM and InterM with high transmissibility days were both moderate (correlation coefficients: 0.53 and 0.55, respectively; both *p* < 0.05). However, as shown in Fig. 5, these associations were dramatically weakened (0.17 and 0.37; both *p* < 0.05) when only counties with a low level of vulnerability were considered. Furthermore, regarding the correlation between IntraM and high transmissibility days across each vulnerability group, the correlation coefficient gradually increased with increasing vulnerability level (0.17–0.68, *p* < 0.05). However, regarding the correlation between InterM and high transmissibility days, the correlation coefficient increased from vulnerability level 1 to vulnerability level 2 and then began to fluctuate by ~0.6 (0.54–0.63, *p* < 0.05) from vulnerability level 3 to vulnerability level 5.

After exploring the associations above, a fixed effect model (Eq. (14)) was employed to estimate the relationship between mobility and *R*_*t*_ for each county. Clearly, the coefficients of IntraM and InterM were significantly positive for all vulnerability levels (*p* < 0.05) (Table 3), implying that an increase in both InterM and IntraM would increase the *R*_*t*._ In other words, both IntraM and InterM accelerated the transmission of COVID-19 in the US during the study period. In addition, in the counties with vulnerability levels between 1 and 4, the coefficients of IntraM and InterM are more equivalent than the coefficients of counties at vulnerability level 5, where the effect of IntraM is almost 2 times larger than that of InterM.

**Table 3:** The coefficients of the fixed effect model for IntraM and InterM across different vulnerability levels.

Vulnerability level	1	2	3	4	5
IntraM	0.07*	0.19***	0.29***	0.22***	0.61***
InterM	0.09***	0.12***	0.18***	0.28***	0.22***

The coefficients of intracounty mobility and intercounty mobility vary over different vulnerability levels and show the generally increasing impact of mobility on COVID-19 transmissibility from level 1 to level 5.

*p < 0.05, **p < 0.01, and ***p < 0.001.

**Fig. 6:**
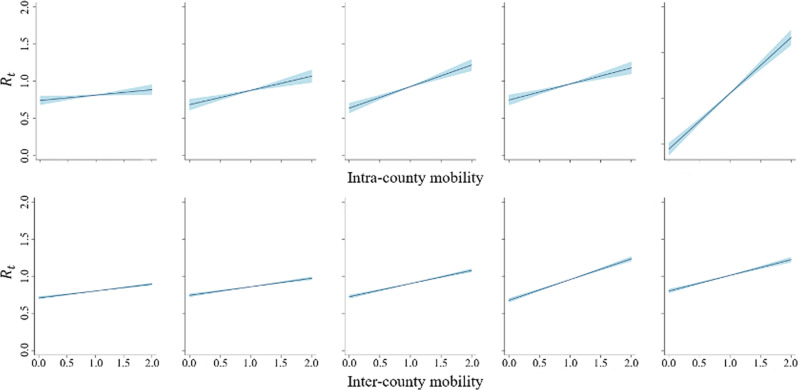
The marginal effects of intracounty mobility (IntraM) and intercounty mobility (InterM) on *R*_*t*_ across different vulnerability levels. The two rows of charts from left to right correspond to vulnerability level 1 to vulnerability level 5.

Furthermore, we examined whether the effects of mobility on *R*_*t*_ are significantly heterogeneous across different vulnerability levels. When designating the effect of IntraM in counties at vulnerability level 1 as a reference, the IntraM coefficients in counties at the other 4 levels were significantly larger (*p* < 0.05) (Table 3). Figure 6 shows the effects of IntraM and InterM on *R*_*t*_ over different vulnerability levels (i.e., 1 to 5 from left to right). Using the group of counties at the highest vulnerability level as a reference, we observed that the effects of IntraM in counties at the other four levels were smaller. In other words, a 25% change in IntraM was associated with a 1.81% (interquartile range (IQR): 0.11–3.51%) change in *R*_*t*_ for the lowest vulnerability level (level 1) but a 15.28% (IQR: 13.35–17.21%) change in *R*_*t*_ for the highest vulnerability level (level 5), which is eight times larger than the former (Table 4). However, for the counties at vulnerability levels 2–4, the changes in the effect of IntraM were marginal. A similar pattern was observed for InterM. The magnitude of the coefficients of InterM increased from 0.09 to 0.28 for counties with a vulnerability level from 1 to 4, with a slight decrease from 0.28 for level 4 to 0.22 for level 5 (Table 3). Additionally, a 25% change in InterM resulted in a 2.31% (IQR: 1.83–2.79%) change in the *R*_*t*_ for level 1 but a 6.98% (IQR: 6.23–7.72%) change in the *R*_*t*_ for level 4.

**Table 4:** The effect of a 25% change in intracounty mobility (IntraM) and intercounty mobility (InterM) on the change in the *R*_*t*_ in percentage form (IQR).

Vulnerability level	*R*_*t*_ changes	
	IntraM change IQR (%)	InterM change IQR (%)
1	1.81 (0.11, 3.51)	2.31 (1.83, 2.79)
2	4.80 (2.89, 6.72)	2.87 (2.36, 3.39)
3	7.25 (5.45, 9.06)	4.43 (3.85, 5.03)
4	5.40 (3.52, 7.27)	6.98 (6.23, 7.72)
5	15.28 (13.35, 17.21)	5.28 (4.48, 6.08)

However, the *F*-test results showed two distinct effects of IntraM and InterM (Table 5). For IntraM, the differences between the coefficients of levels 2–4 were not statistically significant, but the coefficients of level 1 and level 5 were significantly different from those of the other three levels (*p* < 0.05). This result illustrates that the coefficients of IntraM across all 5 vulnerability levels could be divided into three parts—level 1, levels 2–4, and level 5. The differences between the three parts were significant, but the coefficients within each part (only part 2) were not statistically significantly different from each other. Furthermore, for InterM, the difference between the coefficients of level 1 and level 2 was marginal; however, for both levels, the coefficients were significantly different from those of the other three levels (*p* < 0.05). That is, the coefficients of InterM across all 5 vulnerability levels could be divided into two parts, levels 1–2 and levels 3–5. Different from IntraM, there was an inverted U-shaped change in the coefficients in the second part, that is, a slight but significant increase in InterM from level 3 to level 4, as well as a significant decrease from level 4 to level 5.

**Table 5:** Differences in the mobility coefficients between two different vulnerability levels based on analysis of variance (ANOVA) contrasts.

Vulnerability level comparison	Intra-County mobility			Inter-County mobility		
	Difference/95% CI/*F*-Statistic			Difference/95% CI/*F*-Statistic		
2 vs. 1	0.12^*^	(0.02 0.22)	5.23	0.02	(−0.01 0.05)	2.45
3 vs. 1	0.22^***^	(0.12 0.32)	18.51	0.09^***^	(0.05 0.12)	30.3
4 vs. 1	0.14^**^	(0.04 0.24)	7.73	0.19^***^	(0.15 0.22)	106.9
5 vs. 1	0.54^***^	(0.44 0.64)	105.65	0.12^***^	(0.08 0.16)	38.89
3 vs. 2	0.1	(−0.01 0.20)	3.33	0.06^***^	(0.03 0.09)	15.36
4 vs. 2	0.02	(−0.08 0.13)	0.19	0.16^***^	(0.13 0.20)	78.76
5 vs. 2	0.42^***^	(0.31 0.53)	57.18	0.1^***^	(0.06 0.13)	24.47
4 vs. 3	−0.07	(−0.18 0.03)	1.96	0.1^***^	(0.06 0.14)	27.46
5 vs. 3	0.32^***^	(0.22 0.43)	35.62	0.03	(−0.01 0.07)	2.76
5 vs. 4	0.4^***^	(0.29 0.50)	52.12	−0.07^**^	(−0.11–0.02)	34.33

*p < 0.05; **p < 0.01; ***p < 0.001.

## Discussion and conclusion

The COVID-19 global pandemic has severely affected economies and societies worldwide; in this regard, population mobility has been identified as a key factor in shaping transmission dynamics. However, understanding how mobility affects COVID-19 transmissibility in different regions tends to be complex. For this reason, we examined the heterogeneity of the effects of mobility on COVID-19 transmissibility.

We developed a comprehensive CPVI for US counties that includes socioeconomic, natural environment, and health care coverage dimensions. Through our modelling, we found a positive association between the CPVI and the high transmissibility days in US counties. This result suggests that our CPVI captures the intrinsic vulnerability of people who tend to be hit hardest by COVID-19 and therefore bear the greatest burden of the pandemic.

We also assessed the joint effects of the CPVI and population mobility (including both IntraM and InterM) on COVID-19 transmissibility (*R*_*t*_). Mobility is positively related to real-time changes in COVID-19 transmissibility (*R*_*t*_) at all vulnerability levels but to varying degrees. Overall, the more vulnerable a county is, the stronger the influence is; this heterogeneity applies to both IntraM and InterM. The results suggest that the effectiveness of unified policies in restricting mobility may be insufficient, thus calling for “customized” policies based on the vulnerability and mobility characteristics of counties.

We noted that the effect of IntraM on the transmissibility of COVID-19 is greater than that of InterM in the group of most vulnerable counties, thereby necessitating the urgent need to implement mobility reduction policies within these counties. The difference in the effects of IntraM versus InterM may be because within-county contact, which is mostly at the household level, is much more frequent than intercounty contact. We also observed different patterns in the changing mobility effect on transmissibility, which could point to different policies. For example, priority can be given to reopening areas with a lower vulnerability level (such as level 1 and level 2) and subsequently reopening other areas in succession based on the level of vulnerability, depending on the development of the pandemic. Social distancing policies that target reducing the level of social contact within a county could be stricter in areas that have a vulnerability level of 2–4, and more demanding social distancing policies and better support could be advised for areas with a vulnerability level of 5.

Additionally, we found a change in the relationship between the vulnerability level and mobility before and after control measures were launched in the US. Specifically, before the lockdown period, the mobilities of counties belonging to all vulnerability level groups were quite similar; however, after lockdown policies were launched, the vulnerability level groups tended to have lower mobility. This finding is consistent with current policies, suggesting that the policies efficiently reduced the mobility of the most vulnerable groups of people. However, it seems to contradict a previous finding showing that state lockdown policies had a larger effect on income-advantaged groups than on reducing their mobility (Jay et al., 2020). This study uses the proportion of days staying at home or at workplaces all day as the measurement of absolute mobility, and we successfully reproduced the patterns consistent with the abovementioned study using our mobility dataset in terms of household income (Fig. S2). Thus, the disparity results because our study measures relative mobility (compared to a baseline time period) rather than absolute mobility and uses the CPVI rather than household income. By combining the work of Jay et al. with our own, we catch sight of a more complete picture; even though the lockdown policy reduced most of the absolute mobility of the income-advantaged group (who should be regarded as the least vulnerable group), it actually disproportionately affected the most vulnerable group by restricting their nonwork mobility when compared to the reduction in relative mobility. This result also calls attention to the importance of rigorously selecting and precisely measuring the dependent variables in policy evaluation.

Compared with studies that focus solely on one dimension (e.g., income or unemployment only), our study established a comprehensive measurement of vulnerability to the COVID-19 pandemic. We proposed a new composite indicator, namely, CPVI, which emphasizes the COVID-19 risk of socially vulnerable groups by considering the socioeconomic, natural environment, and epidemiological factors. The empirical results suggest that the CPVI captures extremely vulnerable counties in all dimensions included, which provides unique advantages and insights that previous similar studies are unable to capture.

Our study was conducted under several data limitations and assumptions. First, 1118 counties were included in this study. Although accounting for only 35% of all counties in the United States, these counties include 78% of the US population and more than 82% of the cumulative confirmed cases, thus reflecting the true status of the COVID-19 pandemic in the United States. Second, even though fixed effect models were used to minimize the error caused by time-invariant confounders, time-variant confounders such as temperature and people’s knowledge of COVID-19 were not included in the models. Therefore, we also tested another fixed effect model that incorporated time-fixed effects in addition to individual fixed effects. We supposed that the effects of factors that are time variant (but consistent across counties) could be taken into account by the time factor. The results of the time and individual fixed effect models (Tables S2, S3, and Fig. S3) are consistent with the results from our original fixed effect model, even though the coefficient magnitudes of IntraM and InterM at different vulnerability levels change. Third, the incubation period was set as 0 in this study. Given an estimation of the mean incubation period of COVID-19 to be approximately 5 days (Lauer et al., 2020), to test whether our model is robust to the varying incubation periods, we ran our model again with the 5-day incubation length. The results (Tables S4, S5, and Fig. S4) show that the interaction effect of the CPVI and mobility is consistent with and robust to different lengths of the incubation period. Fourth, there might be other factors affecting the relationship between mobility and COVID-19 transmissibility, such as the disease-preventive behaviour adopted by people in social contact. The population’s disease-preventive behaviour, such as avoiding visiting crowded places, directly reduces mobility. Wearing a face mask is also observed to be effective in reducing transmissibility (Cowling et al., 2020). Therefore, disease-preventive behaviour was treated as time-invariant and removed from the calculation process under the assumption that people’s disease-preventive behaviour is more constant compared to the outbreak and the lockdown period (March–June 2020). Fifth, the underlying assumption of using the traditional PCA to compose our CPVI is based on the linear combination of features. However, the relations among those features could be complex. Kernel-PCA may be explored in this regard. Finally, this study was conducted under nonvaccination intervention conditions, which may have some impact on the results of this study when more effective assays are subsequently found and when vaccines are administered and distributed widely. Nevertheless, the method developed in this study can still be applied, although vaccination may change the mobility and transmissibility numbers. Our study also suggests that the distribution of vaccines should assign priority based not only on the age structure but also on other vulnerability factors that tend to be overlooked, such as counties with poor air quality, a little greenery, and/or inadequate medical facilities.

These limitations notwithstanding, our study provides policymakers with a more effective way to explain the transmission of COVID-19, design prevention policies, and carry out additional community-supporting practices. In a paradoxical situation where state governments implement embargo policies that harm regional socioeconomic development and where the blind choice to reopen may exacerbate the spread of COVID-19, our findings should help policymakers develop a policy of batched or gradual reopening.

## Supplementary information

Social vulnerability amplifies the disparate impact of mobility on COVID-19 transmissibility across the United States

## Data Availability

Datasets of confirmed cases of COVID-19 are available at https://usafacts.org/issues/coronavirus/. Population mobility data were obtained from https://github.com/GeoDS/COVID19USFlows, and county attribute data from several organizations are specified in the Methods “County attribute data”. The code for this study is available at https://github.com/huangzhihui-421/Social-vulnerability-amplifies-the-disparate-impact-of-mobility-on-COVID-19-transmissibility-across-.
