# Supplementary material for: Social vulnerability amplifies the disparate impact of mobility on COVID-19 transmissibility across the United States
Source: Humanit Soc Sci Commun. 2022 Nov 24;9(1):415. doi: 10.1057/s41599-022-01437-5 (PMC9702777; doi:10.1057/s41599-022-01437-5)
Supplement: Supplementary file 1 — Social vulnerability amplifies the disparate impact of mobility on COVID-19 transmissibility across the United States [file 41599_2022_1437_MOESM1_ESM.docx]

**Supplementary Materials**

**Social vulnerability amplifies the disparate impact of mobility on COVID-19 transmissibility across the United States**

**S1. Review of social vulnerability index**

Along with the previous literature of vulnerability, scholars construct vulnerability indices pertaining to COVID-19 with consideration of sensitivity, exposure and adaptive capacity (Adger 2006). The divergence in measuring exposure leads to two different ways of selecting variables. One group of scholars follow the traditional framework of variable selection, which is under the mindset that the incidence and the scale of a disaster are independent to the social system, and the number of victims of the event (e.g., a natural hazard, such as flooding and climate change) in a specific area will not affect the possibility of exposure in other areas. Therefore, even in face of the highly transmissible pandemic, they do not incorporate incidence and mortality. The vulnerability index is usually constructed using such a method as sum of the equally weighted percentile rank (Acharya and Porwal 2020, Macharia, Joseph et al. 2020, Snyder and Parks 2020, Welsh, Sinclair et al. 2022), statistical models (Daras, Alexiou et al. 2021), or principal component analysis (Kim and Bostwick 2020, Sarkar and Chouhan 2021).

Another group of scholars regard the incidence and mortality caused by the pandemic as an important signal of higher exposure possibility for others, and therefore incorporate them in the index (Amram, Amiri et al. 2020, Marvel, House et al. 2021, Tiwari, Dadhania et al. 2021). Also, because of the enrichment of the daily recorded data such as mobility, test population, vaccination and so on, more sophisticated models such as machine learning (Tiwari, Dadhania et al. 2021) and the combination of statistical models and machine learning (Marvel, House et al. 2021) are adopted in these studies. Data-driven methods help overcome theoretical restrictions in statistical models and improve the modelling accuracy. However, it then becomes challenging to distinguish if the outstanding explanatory power of the index comes from the comprehensive framework or just because of the inclusion of infected cases/Rt in the index. It is also challenging to differentiate if other social economic or environment variables add noises or explanatory power to the index.

S2. County attribute data selection

We grouped the indicators pre-selected in the dataset into five broad categories based on the different ways in which the data describe social and environmental information.

**S2.1 Demographic dimension**

The indicators of the demographic dimension include the following: being an elderly individual, ethnicity, groups, language, and lower education attainment. First, past sociological research on disasters suggests that older adults face chronic health challenges. Thus, COVID-19 is more likely to threaten the elderly population, and counties with more older individuals may be especially vulnerable (Snyder and Parks 2020). Second, minorities are more likely than whites to live in environmentally polluted neighbourhoods, which increases their propensity to develop environmental health problems such as asthma as well as their subsequent risk of COVID-19 morbidity and even death (Barnes, Grant et al. 2007, Collins 2020, Karaye and Horney 2020, Onder, Rezza et al. 2020). In addition, minorities are more likely to work in the service sector, making social segregation less feasible (Farrell and Venator 2012). Third, crowded conditions and complex transient populations in group quarters prevent effective quarantine measures from being implemented, making it easier for the virus to spread from person to person (Chin, Kahn et al. 2020). Finally, people who do not speak English well and who are not highly educated may lack the knowledge of the COVID-19, and show less trust and compliance to the government guidance (Paul, Steptoe et al. 2021).What is more, the communication gaps due to health literacy and English language proficiency may hinder accurate health information dissemination (Tai, Shah et al. 2021, Zhao, Segalowitz et al. 2021), thereby leading to disproportionate impacts of COVID-19 on linguistic minorities and lower educated groups.

**S2.2 Socio-economic dimension**

The indicators of the socio-economic dimension include income, poverty, and unemployment. The fewer people who live below the poverty line and lower the unemployment rate, the higher the standard of living. High values of these indicators can be used to indicate more vulnerable communities (Day 2006, Chin, Kahn et al. 2020). Thus, areas with large numbers of such people are likely to have higher COVID-19 pandemic risk.

**S2.3 Disease**

Indicators of disease dimensions include the following: smoking, diabetes, coronary heart disease (CHD), and hypertension. People with a history of comorbidities (including CHD, hypertension and diabetes) have an increased risk of COVID-19 infection due to the effects of certain medications, and chronic diseases might also increase mortality (Gu, Chu et al. 2020). Tobacco smoking is known to increase the risk of bacterial and viral respiratory infections. Hence, people with comorbidities, as well as smokers, may be more susceptible to SARS-CoV-2.

**S2.4 Natural environment dimension**

The indicators of the environmental dimension include the following: air pollution and green exposure. Prolonged exposure to air pollution has been shown to cause damage to the human respiratory system (Xing, Xu et al. 2016). COVID-19 is primarily spread through the respiratory tract; thus, air pollution may contribute to the rapid spread of the disease. In addition, living near green spaces can be an effective way to reduce stress and, thus, risk of disease (Banay, James et al. 2019). In the particular period of the COVID-19 pandemic, urban green spaces may be a key factor in maintaining a physically and socially active lifestyle without increasing the risk of infection.

**S2.5 Medical security**

The indicators of the medical security dimension include the following: isolation rooms, hospital beds, and insurance. Using airborne isolation rooms is an effective means of suppressing respiratory pandemics such as COVID-19. Meanwhile, the number of medical beds in a county reflects the capacity of the county to receive patients. However, with the outbreak of COVID-19, several counties are experiencing severe shortages of medical facilities, and diagnosed populations living in counties where health facilities are scarce are likely to seek care in well-equipped areas (Chin, Kahn et al. 2020, Snyder and Parks 2020). Furthermore, people without health insurance may choose not to go to the hospital for treatment because they cannot afford expensive medical care. All of the factors above may increase the COVID-19 pandemic risk.


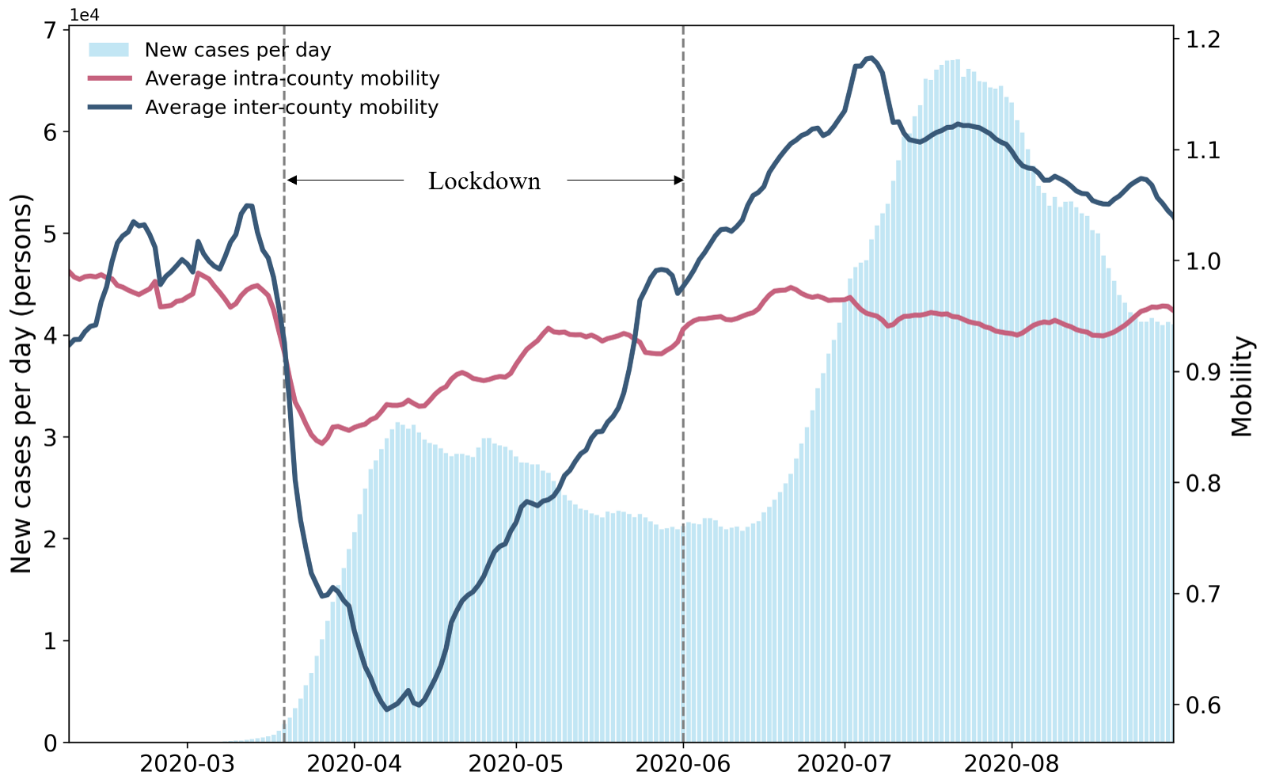


**Fig. S1 COVID-19 pandemic and mobility changes in the US.** The daily new cases, IntraM, InterM, and national lockdown period are presented. New cases per day were calculated by the cumulative number of cases in each county on that day minus the cumulative number of cases in the county on the previous day. All mobility values were the US national average values and were smoothed using a seven-day moving average. The national lockdown period is defined based on when states declare home orders and reopening. California was the first state in the United States to enact a social distancing policy on March 19, other states followed suit, and then, each state had a reopening policy in May. We defined March 19 to June 1 as the lockdown period.


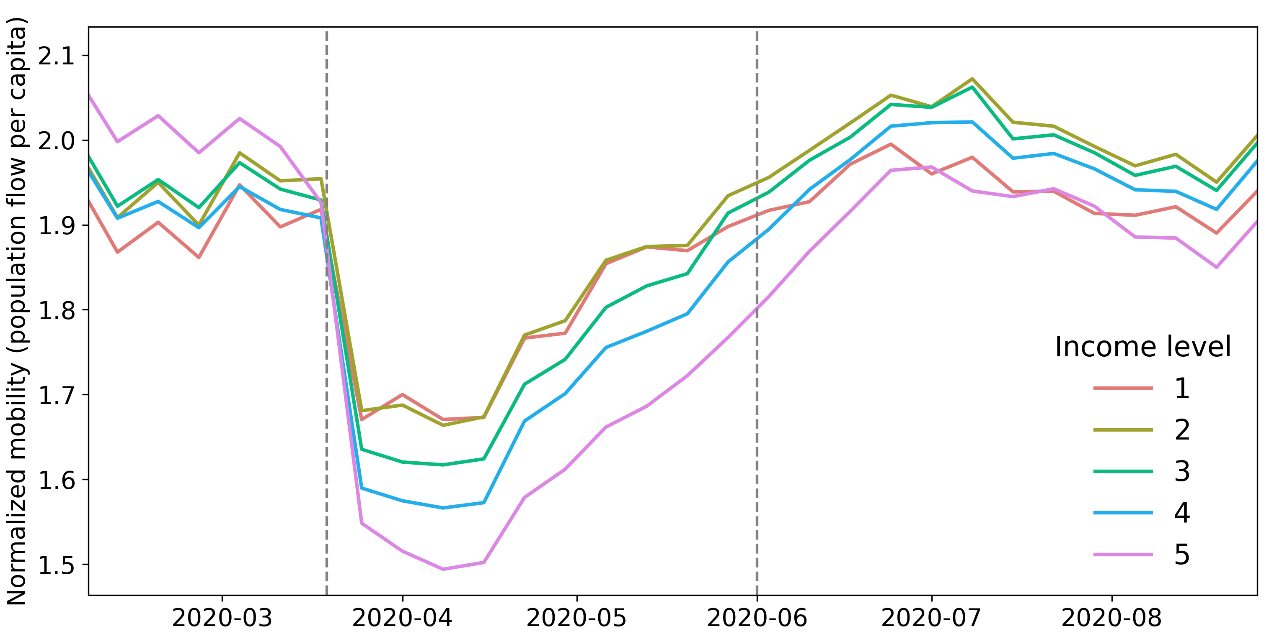


**Fig. S2 Temporal variation of mobility for different income levels.** The income is divided into five levels using quintiles, with level 1 indicating the lowest income. The population flow for each county is normalized using the population size, and outcomes are presented as weekly averages.


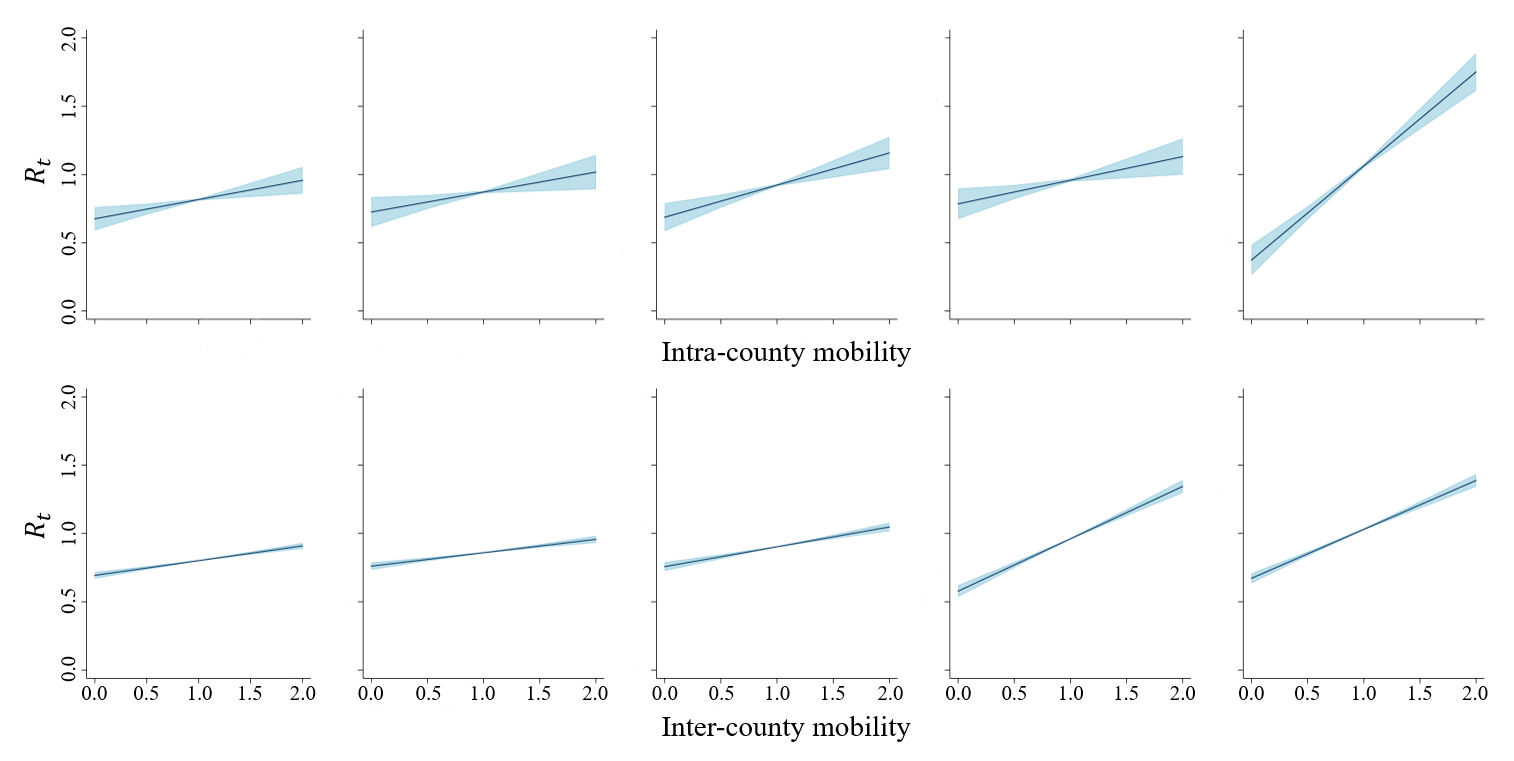


**Fig. S3 The marginal effects of IntraM and InterM on the R_t_ at varying CPVI levels (results of time and individual fixed effect models).** The two rows of charts from left to right correspond to vulnerability level 1 to vulnerability level 5.


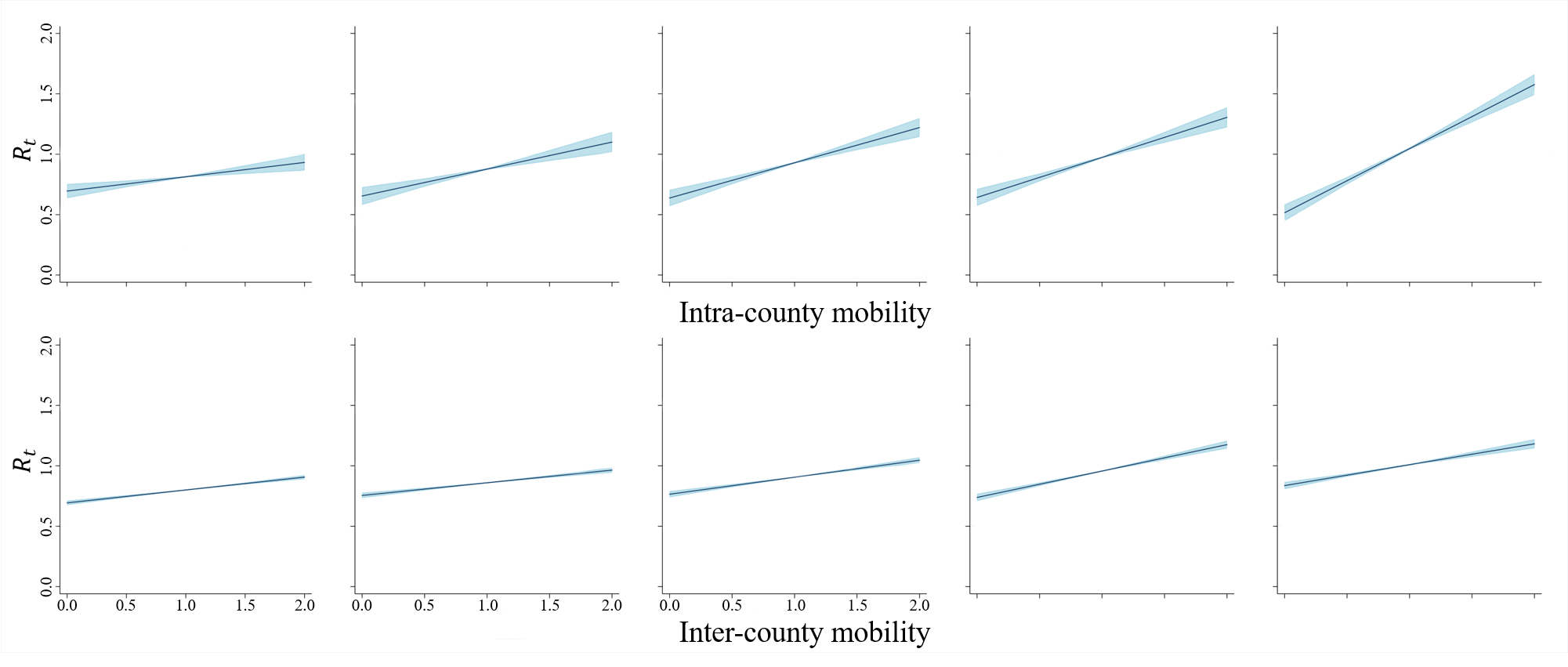


**Fig. S4 The marginal effects of IntraM and InterM on the R_t_ at different CPVI levels (with 5-days timelag of mobility).** The two rows of charts from left to right correspond to vulnerability level 1 to vulnerability level 5.

| **Table S1. County attribute data.** | | | | |
| --- | --- | --- | --- | --- |
| Category | Variable | Description | Direction | Sources |
| Demographic | Elderly | Number of people over 65 years of age | Positive | CDC |
|  | Ethnicity | Number of minorities (all persons except white, non-Hispanic) | Positive | CDC |
|  | Groups | Number of persons in group quarters | Positive | CDC |
|  | Language | Number of persons who speak English “less than well” | Positive | CDC |
|  | Education | Number of persons with no high school diploma | Positive | CDC |
| Socio-economic | Income | Per capita income (PCI) | Negative | CDC |
|  | Poverty | Number of persons below the poverty line | Positive | CDC |
|  | Unemployment | Number of unemployed persons | Positive | CDC |
| Disease | Smoking | Percentage of adult smokers | Positive | CDC |
|  | Diabetes | Percentage of diagnosed diabetes patients | Positive | CDC |
|  | Coronary heart disease (CHD) | Coronary heart disease Hospitalization rate per 1,000 | Positive | CDC |
|  | Hypertension | Hypertension hospitalization rate per 1,000 people | Positive | CDC |
| Natural environment | Air pollution | Means of PM_2.5_ | Positive | CHR |
|  | Green exposure | Mean of the normalized difference vegetation index (NDVI) | Negative | MODIS 13A from GEE |
| Medical security | Isolation rooms | Number of airborne isolation rooms per 100,000 people | Negative | AHRF |
|  | Hospital beds | Number of hospital beds per 100,000 people | Negative | AHRF |
|  | Insurance | Percentage of persons without health insurance | Negative | CDC |
| Note: CDC stands for Centers for Disease Control and Prevention, CHR County Health Ranking & Roadmaps, GEE Google Earth Engine, and AHRF Area Health Resources Files. | | | | |

**Table S2. The coefficients of time and individual fixed effect models for IntraM and InterM under varying vulnerability levels (p < 0.05).**

| Vulnerability level | 1 | 2 | 3 | 4 | 5 |
| --- | --- | --- | --- | --- | --- |
| IntraM | 0.14 | 0.15 | 0.24 | 0.17 | 0.69 |
| InterM | 0.11 | 0.10 | 0.15 | 0.38 | 0.36 |

**Table S3. Differences in the mobility coefficients between vulnerability levels using time and individual fixed effect models.**

| Vulnerability Level Comparison | Intra-County Mobility | | | Inter-County Mobility | | |
| --- | --- | --- | --- | --- | --- | --- |
|  | Difference/95% CI/F-Statistic | | | Difference/95% CI/F-Statistic | | |
| 2 vs. 1 | 0.15^**^ | (0.05 0.25) | 8.56 | 0.01 | (-0.02 0.04) | 0.71 |
| 3 vs. 1 | 0.3^***^ | (0.20 0.39) | 36.24 | 0.06^***^ | (0.03 0.09) | 15.65 |
| 4 vs. 1 | 0.19^***^ | 0.09 0.29 | 14.43 | 0.18^***^ | (0.14 0.21) | 101.18 |
| 5 vs. 1 | 0.52^***^ | (0.41 0.61) | 102.73 | 0.13^***^ | (0.09 0.16) | 46.56 |
| 3 vs. 2 | 0.15^**^ | (0.04 0.25) | 7.98 | 0.05^**^ | (0.02 0.08) | 9.46 |
| 4 vs. 2 | 0.04 | (-0.06 0.14) | 0.63 | 0.17^***^ | (0.13 0.20) | 84.34 |
| 5 vs. 2 | 0.37^***^ | (0.26 0.47) | 47.02 | 0.12^***^ | (0.08 0.15) | 36.83 |
| 4 vs. 3 | -0.1^*^ | (-0.20 0.00) | 4.18 | 0.12^***^ | (0.08 0.15) | 40.05 |
| 5 vs. 3 | 0.22^***^ | (0.12 0.32) | 18.05 | 0.07^***^ | (0.03 0.11) | 11.96 |
| 5 vs. 4 | 0.32^***^ | (0.22 0.43) | 37.91 | -0.05^*^ | (-0.09 -0.01) | 5.51 |

Note: * p < 0.05, ** p < 0.01, *** p < 0.001.

**Table S4. The coefficients of fixed effect models (with 5 days timelag of mobility) for IntraM and InterM under varying vulnerability levels (p < 0.05).**

| Vulnerability level | 1 | 2 | 3 | 4 | 5 |
| --- | --- | --- | --- | --- | --- |
| IntraM | 0.12 | 0.22 | 0.29 | 0.33 | 0.53 |
| InterM | 0.11 | 0.10 | 0.14 | 0.22 | 0.17 |

**Table S5. Differences in the mobility coefficients between vulnerability levels using fixed effect models (with 5 days timelag of mobility).**

| Vulnerability Level Comparison | Intra-County Mobility | | | Inter-County Mobility | | |
| --- | --- | --- | --- | --- | --- | --- |
|  | Difference/95% CI/F-Statistic | | | Difference/95% CI/F-Statistic | | |
| 2 vs. 1 | 0.10^*^ | (0.00 0.21) | 4.05 | 0.00 | (-0.03 0.03) | 0.02 |
| 3 vs. 1 | 0.17^**^ | (0.07 0.27) | 11.80 | 0.03^*^ | (0.00 0.06) | 4.75 |
| 4 vs. 1 | 0.21^***^ | (0.11 0.31) | 17.17 | 0.11^***^ | (0.08 0.15) | 37.49 |
| 5 vs. 1 | 0.41^***^ | (0.31 0.51) | 62.75 | 0.07^***^ | (0.03 0.10) | 11.76 |
| 3 vs. 2 | 0.07 | (-0.04 0.17) | 1.63 | 0.04^*^ | (0.00 0.07) | 4.93 |
| 4 vs. 2 | 0.11^*^ | (0.00 0.21) | 3.94 | 0.11^***^ | (0.08 0.15) | 36.89 |
| 5 vs. 2 | 0.31^***^ | (0.20 0.42) | 31.11 | 0.07^**^ | (0.03 0.11) | 11.89 |
| 4 vs. 3 | 0.04 | (-0.06 0.14) | 0.57 | 0.08^***^ | (0.04 0.12) | 15.57 |
| 5 vs. 3 | 0.24^***^ | (0.13 0.34) | 20.00 | 0.03 | (-0.01 0.07) | 2.45 |
| 5 vs. 4 | 0.20^***^ | (0.09 0.31) | 13.45 | -0.05^*^ | (-0.09 -0.00) | 3.93 |

Note: * p < 0.05, ** p < 0.01, *** p < 0.001.

References

Acharya, R. and A. Porwal (2020). "A vulnerability index for the management of and response to the COVID-19 epidemic in India: an ecological study." The Lancet Global Health **8**(9): e1142-e1151.

Adger, W. N. (2006). "Vulnerability." Global environmental change **16**(3): 268-281.

Amram, O., S. Amiri, R. B. Lutz, B. Rajan and P. Monsivais (2020). "Development of a vulnerability index for diagnosis with the novel coronavirus, COVID-19, in Washington State, USA." Health & place **64**: 102377.

Banay, R. F., P. James, J. E. Hart, L. D. Kubzansky, D. Spiegelman, O. I. Okereke, J. D. Spengler and F. Laden (2019). "Greenness and depression incidence among older women." Environmental health perspectives **127**(2): 027001.

Barnes, K. C., A. V. Grant, N. N. Hansel, P. Gao and G. M. Dunston (2007). "African Americans with asthma: genetic insights." Proceedings of the American Thoracic Society **4**(1): 58-68.

Chin, T., R. Kahn, R. Li, J. T. Chen, N. Krieger, C. O. Buckee, S. Balsari and M. V. Kiang (2020). "US county-level characteristics to inform equitable COVID-19 response." MedRxiv.

Collins, S. (2020). "The Trump administration blames Covid-19 black mortality rates on poor health. It should blame its policies." Vox.

Daras, K., A. Alexiou, T. C. Rose, I. Buchan, D. Taylor-Robinson and B. Barr (2021). "How does vulnerability to COVID-19 vary between communities in England? Developing a small area vulnerability index (SAVI)." J Epidemiol Community Health **75**(8): 729-734.

Day, K. (2006). "Active living and social justice: planning for physical activity in low-income, black, and Latino communities." Journal of the American Planning Association **72**(1): 88-99.

Farrell, J. and J. Venator (2012). "Paid sick days work for US employees and employers." Center for American Progress.

Gu, T., Q. Chu, Z. Yu, B. Fa, A. Li, L. Xu, R. Wu and Y. He (2020). "History of coronary heart disease increases the mortality rate of coronavirus disease 2019 (COVID-19) patients: A nested case-control study based on publicly reported confirmed cases in Mainland China." medRxiv.

Karaye, I. M. and J. A. Horney (2020). "The impact of social vulnerability on COVID-19 in the US: an analysis of spatially varying relationships." American journal of preventive medicine **59**(3): 317-325.

Kim, S. J. and W. Bostwick (2020). "Social vulnerability and racial inequality in COVID-19 deaths in Chicago." Health education & behavior **47**(4): 509-513.

Macharia, P. M., N. K. Joseph and E. A. Okiro (2020). "A vulnerability index for COVID-19: spatial analysis at the subnational level in Kenya." BMJ global health **5**(8): e003014.

Marvel, S. W., J. S. House, M. Wheeler, K. Song, Y.-H. Zhou, F. A. Wright, W. A. Chiu, I. Rusyn, A. Motsinger-Reif and D. M. Reif (2021). "The COVID-19 Pandemic Vulnerability Index (PVI) Dashboard: Monitoring county-level vulnerability using visualization, statistical modeling, and machine learning." Environmental Health Perspectives **129**(1): 017701.

Onder, G., G. Rezza and S. Brusaferro (2020). "Case-fatality rate and characteristics of patients dying in relation to COVID-19 in Italy." Jama **323**(18): 1775-1776.

Paul, E., A. Steptoe and D. Fancourt (2021). "Attitudes towards vaccines and intention to vaccinate against COVID-19: Implications for public health communications." The Lancet Regional Health-Europe **1**: 100012.

Sarkar, A. and P. Chouhan (2021). "COVID-19: District level vulnerability assessment in India." Clinical epidemiology and global health **9**: 204-215.

Snyder, B. F. and V. Parks (2020). "Spatial variation in socio-ecological vulnerability to Covid-19 in the contiguous United States." Health & place **66**: 102471.

Tai, D. B. G., A. Shah, C. A. Doubeni, I. G. Sia and M. L. Wieland (2021). "The disproportionate impact of COVID-19 on racial and ethnic minorities in the United States." Clinical Infectious Diseases **72**(4): 703-706.

Tiwari, A., A. V. Dadhania, V. A. B. Ragunathrao and E. R. Oliveira (2021). "Using machine learning to develop a novel COVID-19 Vulnerability Index (C19VI)." Science of The Total Environment **773**: 145650.

Welsh, C. E., D. R. Sinclair and F. E. Matthews (2022). "Static socio-ecological COVID-19 vulnerability index and vaccine hesitancy index for England." The Lancet Regional Health-Europe **14**: 100296.

Xing, Y.-F., Y.-H. Xu, M.-H. Shi and Y.-X. Lian (2016). "The impact of PM2. 5 on the human respiratory system." Journal of thoracic disease **8**(1): E69.

Zhao, Y., N. Segalowitz, A. Voloshyn, E. Chamoux and A. G. Ryder (2021). "Language barriers to healthcare for linguistic minorities: The case of second language-specific health communication anxiety." Health communication **36**(3): 334-346.
